# Brain network reorganization differs in response to stress in rats genetically predisposed to depression and stress-resilient rats

**DOI:** 10.1038/tp.2016.233

**Published:** 2016-12-06

**Authors:** N Gass, R Becker, A J Schwarz, W Weber-Fahr, C Clemm von Hohenberg, B Vollmayr, A Sartorius

**Affiliations:** 1 Department of Neuroimaging, Research Group Translational Imaging, Central Institute of Mental Health, Medical Faculty Mannheim, Heidelberg University, Mannheim, Germany; 2 Department of Psychological and Brain Sciences, Indiana University, Bloomington, IN, USA; 3 Department of Radiology and Imaging Sciences, Indiana University, Indianapolis, IN, USA; 4 Department of Psychiatry and Psychotherapy, Central Institute of Mental Health, Medical Faculty Mannheim, Heidelberg University, Mannheim, Germany

## Abstract

Treatment-resistant depression (TRD) remains a pressing clinical problem. Optimizing treatment requires better definition of the specificity of the involved brain circuits. The rat strain bred for negative cognitive state (NC) represents a genetic animal model of TRD with high face, construct and predictive validity. Vice versa, the positive cognitive state (PC) strain represents a stress-resilient phenotype. Although NC rats show depressive-like behavior, some symptoms such as anhedonia require an external trigger, i.e. a stressful event, which is similar to humans when stressful event induces a depressive episode in genetically predisposed individuals (gene–environment interaction). We aimed to distinguish neurobiological predisposition from the depressogenic pathology at the level of brain-network reorganization. For this purpose, resting-state functional magnetic resonance imaging time series were acquired at 9.4 Tesla scanner in NC (*N*=11) and PC (*N*=7) rats before and after stressful event. We used a graph theory analytical approach to calculate the brain-network global and local properties. There was no difference in the global characteristics between the strains. At the local level, the response in the risk strain was characterized with an increased internodal role and reduced local clustering and efficiency of the anterior cingulate cortex (ACC) and prelimbic cortex compared to the stress-resilient strain. We suggest that the increased internodal role of these prefrontal regions could be due to the enhancement of some of their long-range connections, given their connectivity with the amygdala and other default-mode-like network hubs, which could create a bias to attend to negative information characteristic for depression.

## Introduction

Treatment-resistant depression (TRD) remains a pressing clinical problem responsible for long-term disability and posing a therapeutic challenge. A better definition of the specificity of the brain circuits involved in TRD would be beneficial for the optimization and development of treatments. Most of the neuroimaging studies in depressed individuals represent snapshots of the brain activity collected once at any given point during depression, thus making it difficult to distinguish neurobiological predisposition from the depressogenic pathology. Understandably, it is difficult to trait an individual history of depressed individuals, performing longitudinal experiments at the 'at-risk' stage and during the depressive episode, in addition leaving a patient unmedicated. This question can be more suitably addressed using animal models. Two rat strains (congenital learned helpless (cLH) and congenital non-learned helpless (cNLH)) were bred based on a Seligman's hypothesis that learned helplessness, a depression-like condition, is induced by uncontrollable stress in individuals with a predisposition.^1^ The cLH strain represents a genetic animal model of TRD with high face, construct and predictive validity.^2, 3, 4^ In contrast, the cNLH strain represents a stress-resilient phenotype. This established genetic strain of cLH rats demonstrate congenital helpless behavior, as well as deficient reward perception,^5^ depressive-like cognitive bias,^2, 6^ treatment resistance (to antidepressant and to electroconvulsive shock therapy)^7^ and biochemical changes such as higher glutamate/GABA ratios in the hippocampus and prefrontal cortex.^7, 8^ Increased glutamate and reduced GABA levels are also observed in depressive patients and could reflect depression-like symptomatology.^9^ Glutamate injection into the rat's frontal cortex is enough to induce learned helplessness.^10^ In addition, the metabolism of the lateral habenula was found to be hyperactive in cLH rats,^11^ and its pharmacological inhibition by GABA agonist muscimol reduces learned helplessness behavior in cLH rats^12^ similarly to the deep-brain stimulation of this region in TRD.^13^

After more than 20 generations, it was noted that cLH rats display helplessness without exposure to uncontrollable shock. Therefore, we started behavioral testing without inescapable shock exposure in order to minimize suffering of the rats. After the procedural change, the cLH strain was named strain bred for negative cognitive state (NC) and the cNLH - as strain bred for positive cognitive state (PC). These two strains differ in susceptibility to develop stress-escape behavior in operant boxes when exposed to escapable foot shock: NC strain exhibits an escape deficit expressed as increased number of failures to terminate the shock and an increased latency to stop the trials, whereas the PC strain almost completely acquires stress control.^14^

It has been shown that, although the NC rats show depressive-like behavior, some symptoms such as anhedonia require an external trigger, i.e. a stressful event (for example, foot shocks within the helplessness test condition) for their manifestation.^5^ Similarly to humans, stress interacts with the genetic predisposition to depressive-like behavior (gene–environment interaction) and triggers anhedonia and deficient reward perception in NC rats.^5^

In the current study we aimed to discriminate the difference in response to stress in the risk and depressive-like endophenotypes at the level of brain-network reorganization using a graph theoretical approach, which gives a detailed picture of the changes in the brain. For this purpose, we scanned the PC and NC rats before and after a helplessness test. On the basis of the previous report,^5^ the NC rats from the first functional magnetic resonance imaging (fMRI) measurement would represent the risk phenotype, from the second - the depressive-like behavior phenotype, whereas the PC rats would represent the resilient phenotype. We conducted the whole-brain exploratory analysis to determine which regions would be stress-reactive, that is, would change their nodal role in response to stress differently in the risk phenotype compared to the resilient phenotype.

## Materials and methods

### Animals

Sprague–Dawley rats were selected for subsequent generations on the basis of their susceptibility to develop stress-escape behavior in operant boxes. 12 NC and 9 PC rats (eightieth generation; 262–394 g; 8-week old) participated in the fMRI measurements. Animals were housed in plastic cages 38 × 20 × 59 cm, 4 animals per cage, in a room maintained at constant temperature of 22 °C and 12- h light–dark cycle. Food and water were provided *ad libitum*.

As the study is exploratory, no formal power or sample size estimation was performed; however, the group sizes (*N*=9–12 per group) are toward the high end of the range typically used in rat fMRI experiments.

All experiments were conducted according to the regulations covering animal experimentation within the European Union (European Communities Council Directive 86/609/EEC) and within the German Animal Welfare Act. The experiments were approved by the German animal welfare authorities (Regierungspräsidium Karlsruhe).

### MRI acquisition

The experiments were conducted at a 94/20 Bruker Biospec MRI scanner (9.4 Tesla; Bruker BioSpec, Ettlingen, Germany) with Avance III hardware, BGA12S gradient system (maximum strength 705 mT m^−1^) and Paravision 6 software (Bruker BioSpec). The linear whole-body volume transmitter coil combined with an anatomically shaped four-channel receive-only coil array for the rat brain was used for transmission and reception. The anesthetic regime was the same as described previously.^15^ In brief, the rats were initially anesthetized with 4% isoflurane (Baxter Deutschland, Unterschleissheim, Germany) in a mixture of N_2_ (70%) and O_2_ (30%); after positioning in the scanner, the isoflurane level was reduced to 2.5% and medetomidine (Domitor, Janssen-Cilag, Neuss, Germany) was administered (first a 0.5 ml bolus, 0.07 mg kg^−1^ subcutaneously (s.c.), and, afterward, continuous infusion at a 0.28 mg kg^−1^ per hour rate). Isoflurane administration was discontinued within 10 min before start of the continuous medetomidine infusion.

**Figure 1:**
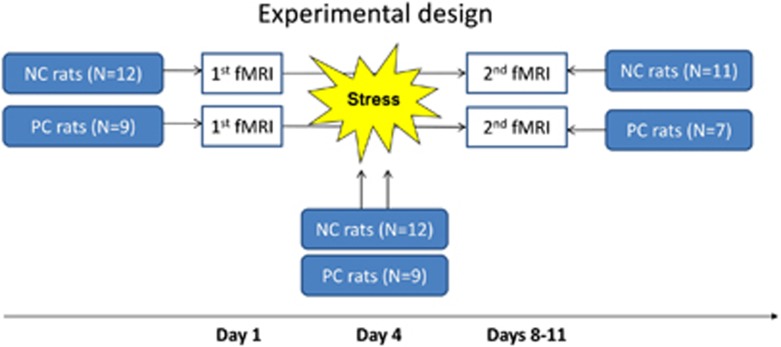
An overview of the experimental design displaying three experimental conditions along the timescale. The first fMRI measurement was followed by the behavioral stress and by the second fMRI experiment. For each condition, the number of animals participating in the experiment is indicated. fMRI, functional magnetic resonance imaging; NC, negative cognitive state; PC, positive cognitive state.

The animals were measured twice—before and after the stress-inducing behavioral test (Figure 1 depicts the experimental design). The second measurement included *N*=11 of NC rats and *N*=8 of PC rats. The number of rats was lower because of the technical defects in the coil (the coil elements had to be replaced), and the distorted data had to be excluded.

Randomization was used to compose the daily schedules of fMRI measurements according to the group (PC/NC) and time of day. During the experiment, the investigator was blinded to the group allocation.

The MRI acquisition protocol included a FieldMap, a resting state-fMRI (rs-fMRI) time series and a three-dimensional structural data set. Rs-fMRI was acquired following stabilization of physiological parameters (20 min after beginning of the continuous medetomidine administration).

The rs-fMRI time series were acquired using a T2*-weighted echo-planar imaging–free induction decay sequence with the following parameters: repetition time/echo time (TR/TE) 1500/17.5 ms, flip angle 60°, 30 coronal slices (ascending slice order), field of view 35 × 35 mm^2^, voxel dimension 0.365 mm, slice thickness 0.5 mm and 340 acquisitions over 8.5 min.

A three-dimensional double-gradient echo FieldMap sequence (TR=20 ms, short TE=1.7 ms, long TE=5.7 ms) was acquired before EPI. The measured field values were later used in pre-processing to calculate the geometric distortions.

Structural data were acquired using a T2-weighted rapid acquisition with refocused echoes (RARE) sequence with the following parameters: RARE factor 16, TR/TE 1200/50 ms, flip angle 180°, the voxel dimension 0.15 mm and acquisition time 23 min.

Breathing and cardiac signals were acquired using a respiration pad placed beneath the chest (Small Animal Instruments, Stony Brook, NY, USA) and a pulse oximeter attached to the hindpaw, respectively. We used the signal breakout module (Small Animal Instruments) and the four-channel recorder (Velleman, Gavere, Belgium) to record signals (10 ms resolution).

### Animal behavior testing

Behavioral test consisted of escape paradigm where an animal could avoid electrical foot shocks by pressing a lever. In all, 12 NC and 9 PC rats participated in the test.

Experiments were performed between 0800 and 1200 hours in operant conditioning chambers with inside dimensions of 48.5 × 30 × 21.5 cm. The floor was constructed of steel rods 6 mm in diameter and 20 mm spaced apart. On one side of the boxes, there was the 35 × 35 mm lever. The experiment was controlled using an IBM-compatible 4/86 computer. Boxes, shock generator and controlling program were obtained from TSE (Bad Homburg, Germany).

Testing consisted of 10 trials of 0.8 mA current lasting 60 s each (intertrial time 24 s), if the animal did not stop the current by pressing the lever. The latency to press the lever was recorded. The following parameters were analyzed: the sum of latencies for trials 3–10 and the number of trials not stopped within the first 20 s (deficit pattern).^16^ We chose the sum of latencies from trials 3 to 10, as in the first two trials the rats react with hyperlocomotive agitation.^16^

A nonparametric Mann–Whitney *U-*test was used to calculate the differences of deficit pattern and sum of latencies between the NC and PC groups due to the non-normal distribution of the data. *P*=0.05 was used as the threshold for statistical significance.

### Image pre-processing

The pre-processing was done as described previously.^17^ In brief, EPI time series were corrected for magnetic field (B0) inhomogeneities using the FieldMap data (SPM8, http://www.fil.ion.ucl.ac.uk/spm/software/spm8), and the estimated movement parameter vectors were regressed out of each voxel (FSL, version 4.1. http://www.fmrib.ox.ac.uk/fsl) after the realign and unwarp step (SPM8). Afterward, respiratory and cardiac signals were filtered out from the imaging data using the Aztec software,^18^ followed by slice-timing correction and spatial normalization to the rat brain template in Paxinos space with co-registered anatomical atlas^19^ (SPM8). The time course from cerebrospinal fluid (CSF) was filtered from the normalized images: first, a CSF mask was created for each data set, and then its time course was extracted and filtered out (FSL). In the end, the images were band-pass-filtered (0.01–0.1 Hz; Analysis of Functional Neuroimages (AFNI) version 2).^20^

### Graph theoretical analysis

The analysis included only the data with good quality. As mentioned above, due to the technical defects in the coil, we had to exclude 1 NC and 2 PC rats; therefore, the final number for the analysis comprised 11 NC and 7 PC rats.

**Figure 2:**
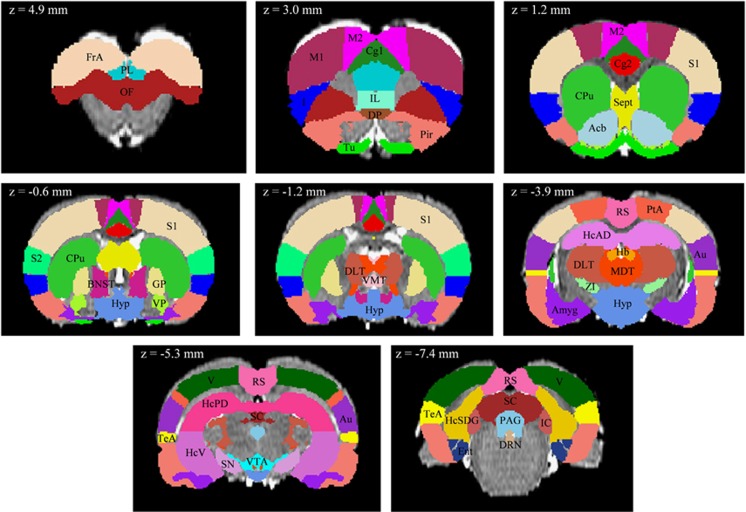
Location of 43 regions of interest used in the graph analysis. Coordinates along the anteroposterior *z*-bregma axis are given in the stereotactic space of Paxinos and Watson.^47^ Acb, nucleus accumbens; Amyg, amygdala; Au, auditory cortex; BNST, bed nucleus of stria terminalis; Cg1, cingulate cortex, area 1; Cg2, cingulate cortex, area 2; CPu, caudate–putamen; DLT, dorsolateral thalamus; DP, dorsal peduncular cortex; DRN, dorsal raphe nuclei; Ent, entorhinal cortex; FrA, frontal association cortex; GP, globus pallidus; Hb, habenula; HcAD, hippocampus, anterodorsal; HcPD, hippocampus, posterodorsal; HcSDG, hippocampus, subiculum and dentate gyrus; HcV, hippocampus, ventral; Hyp, hypothalamus; I, insular cortex; IC, inferior colliculus; IL, infralimbic cortex; M1, primary motor cortex; M2, secondary motor cortex; MDT, midline dorsal thalamus; OF, orbitofrontal cortex; PAG, periaqueductal gray; Pir, piriform cortex; PL, prelimbic cortex; PtA, parietal association cortex; RS, retrosplenial cortex; S1, primary somatosensory cortex; S2, secondary somatosensory cortex; SC, superior colliculus; Sept, septum; SN, substantia nigra; TeA, temporal association cortex; Tu, olfactory tubercle; V, visual cortex; VMT, ventromedial thalamus; VP, ventral pallidum; VTA, ventral tegmental area; ZI, zona incerta.

Functional connectivity graphs were created by correlating the time series of 43 bilateral brain regions pairwise. These regions were defined using Schwarz atlas^19^ and covered the whole brain (Figure 2).

Several lines of work including our group have previously shown the presence of default-mode-like network (DMN) in anesthetized rodents.^21, 22, 23, 24^ Further on, we will use the term DMN, which in rats includes the orbital, prelimbic, dorsal anterior cingulate, retrosplenial cortices and hippocampal regions. The rat dorsal anterior cingulate cortex (ACC) corresponding to the human ACC (area 24) would be part of the DMN in rodent brain similarly to humans.^23^

Edge (connection) weights were determined by all-pairs Pearson correlation matrix and were normalized by maximum correlation, resulting in fully connected weighted undirected graphs for each subject and measurement time. These were converted into weighted networks at various sparsity levels by thresholding such way that a fixed percentage of edges was retained. First, we selected the threshold range from 1 to 80%, with a 1% step. Then, the thresholds were chosen to range from 10% usually used in graph analysis in depression studies;^25, 26^ we have also observed higher s.d.'s for several graph metrics at the thresholds below 10%, thus confirming it as a reasonable value. The upper value was 59%, which was the highest threshold avoiding occurrence of negatively weighted edges in our data. The correlation matrices were analyzed using version 2015-01-25 of Brain Connectivity Toolbox.^27^ To control for possible differences in overall connection strength, for each network, reference random networks were generated using a resampling algorithm to obtain randomly structured networks with preserved degree distribution and approximately preserved strength distribution^28^ (100 random networks were created for averaging for each subject, sparsity level and measurement time).

Modular partitions of the individual networks were detected using the Louvain algorithm. For each individual network, the partitioning procedure was run 100 times. From these 100 partitions, we calculated an intramodule likelihood matrix for each subject. The average intramodule likelihood matrix for all subjects was partitioned (using the Louvain algorithm) to reach a robust modular structure.

Five global metrics were computed for each network:

- Global clustering coefficient was calculated as the average of all nodal clustering coefficients.
- Characteristic path length represents the global average of the graph distance matrix and is related to the efficiency of information transfer within a network (the shorter the path length, the more efficient a network is). It was calculated as the average shortest path length.
- Small-worldness index is the ratio of clustering coefficient to characteristic path length. A small-world network has an organization, which is intermediate between randomness and order: it maintains a high level of clustering (higher than in a random network) and short path length (almost as short as in a random network).
- Global efficiency was computed as the average inverse shortest path length. It captures the extent of information propagation in a network. Global efficiency and characteristic path length measure functional integration, that is, the ability to rapidly combine specialized information from all over the brain.
- Local efficiency was computed as the average of local efficiency values for all nodes.

For each network and sparsity level, eight local graph metrics were computed.

- Degree was defined as the number of adjacent edges connecting a given node to other nodes. It is the most substantial network measure as other metrics are built on it.
- Strength was calculated as the sum of weights of edges connected to the node and can be seen as an extension of degree.
- The path length between two nodes is the minimum connection length (that is, inverse edge weight) that must be traveled to reach one node from the other; it was computed as average of path lengths to all other nodes.
- Betweenness centrality is the percentage of all shortest paths in the network containing the given node. Nodes with high values of betweenness centrality participate in a large number of shortest paths.
- Clustering coefficient was calculated as the number of edges existing between the nodal neighbors, normalized to the maximum number of possible edges. It describes density of edges between the node neighbors; a high level of clustering provides resilience against random perturbations.
- Participation index is the ratio of intermodule strength to the total nodal strength. The participation index is high for nodes that serve as connectors between modules and low for nodes embedded within the module. Participation coefficients were calculated using the group-level partition for each subject individually.
- Local efficiency was calculated as global efficiency computed on a node's neighborhood. It is related to the clustering coefficient and describes the extent of information transfer within the vicinity of the node.
- Local efficiency was normalized (divided) by the global efficiency and this ratio was named *LEGE*.

Network parameter values were integrated over the range of thresholds to calculate the area under the curve (AUC) for each metric. This focuses the analysis toward identifying systematic effects that are not strongly dependent on a specific threshold level, and provides a summarized characterization of brain networks independent of single threshold selection. Groups were compared based on AUC parameters.

A two-way repeated measurements ANOVA was used to calculate the differences of functional network properties taking into account the three following effects: (1) the effect of the time point (the first fMRI measurement versus the second fMRI measurement), (2) the effect of the group (the PC versus the NC), (3) the effect of the interaction between (1) and (2) (with *P*=0.05 used as the threshold for statistical significance). The results were corrected for multiple comparisons over all regions using the false discovery rate (FDR) procedure (*q*<0.1). The effect of the interaction of time and group would answer the question of our study. Therefore, to detect the direction of the effect we conducted a two-sample *t*-test. First we calculated the differences of functional network properties between the first and second session on the individual level. The difference was calculated by subtracting the values of the first measurement from the second measurement for each global and local metrics. The group effect on these differences was assessed by two-sample *t*-test of 11 NC versus 7 PC rats (with *P*=0.05 used as the threshold for statistical significance). The results were corrected for multiple comparisons over all regions using the FDR procedure (*q*<0.1).

## Results

### Behavioral results

**Figure 3:**
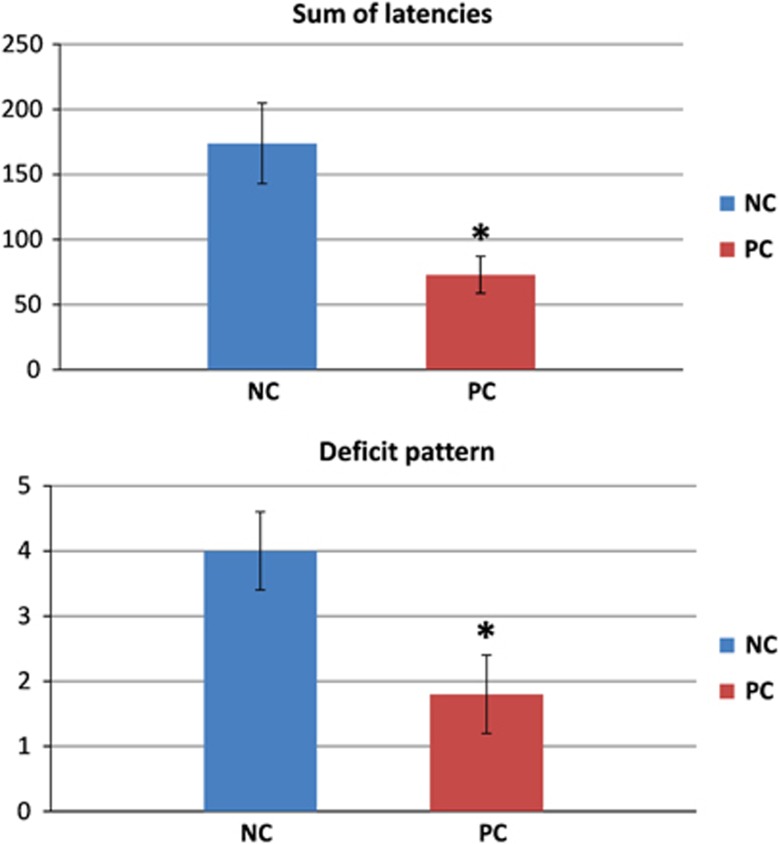
Illustration of the significant differences between the PC and NC rats in the sum of latencies (top) and the deficit pattern (bottom) values in the stress-inducing behavioral test (asterisk (*) indicates statistically significant changes (*P*<0.05)). NC, negative cognitive state strain; PC, positive cognitive state strain.

The sums of latencies to stop the trial were significantly higher for NC rats compared to PC rats (*P*=0.006) similarly to the previous report:^16^ 173.8±30.8 s for the NC and 73.0±14.3 s for the PC rats (Figure 3).

The number of trials not terminated within the first 20 s of a trial (deficit pattern) significantly differed between the groups (*P*=0.018): 4.0±0.6 for the NC and 1.8±0.6 for the PC rats (Figure 3).

### Graph theory analysis

**Figure 4:**
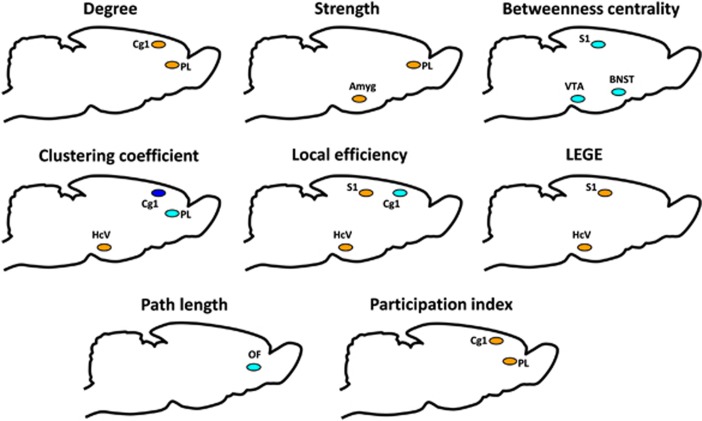
Schematic overview of the significant differences between the PC and NC rats in the graph analysis local metrics. The brain regions are represented as differently colored circles, where light-blue color depicts the reduction of the values in the NC rats compared to PC rats, orange – an increase. Dark-blue color depicts those values which survived FDR correction (q<0.1) and were decreased in the NC rats compared to PC rats. The abbreviations are the same as for Figure 2. FDR, false discovery rate; NC, negative cognitive state strain; PC, positive cognitive state strain.

Here we discuss only the results of the two-sample *t*-test on the group differences since they correspond to ANOVA results on interaction between the group and the time point and give information about the direction of the effect. Figure 4 and Supplementary Material S1 present the results of the two-sample *t*-test. All the ANOVA results are presented in Supplementary Material S2.

### Global results

There were no differences in the network global parameters between the NC and PC groups in their response to stress (Supplementary Material S1).

### Local results

The strongest changes occurred in the dorsal ACC (cingulate cortex area 1): compared to the PC strain, the NC strain had decreased clustering coefficient (*P*<0.05; FDR-corrected). Other nominally significant changes (*P*<0.05; not FDR-corrected) for this region included increased degree, participation index and reduced local efficiency.

The other two regions where several nominally significant changes (not FDR-corrected) occurred were the prelimbic cortex (increased degree, strength and participation index, decreased clustering coefficient in the NC rats), ventral hippocampus (increased clustering coefficient, local efficiency and LEGE) and somatosensory cortex (decreased betweenness centrality, increased local efficiency and LEGE).

## Discussion

There was no difference in the global network characteristics between the risk (NC) and resilient (PC) phenotypes in their response to stress. However, in the local metrics there were several differences between the risk and resilient groups demonstrating reorganization of the nodal roles for the DMN prefrontal hubs, ventral hippocampus and somatosensory cortex. Compared with the resilient strain, the risk strain responded to stress by increasing the internodal roles of the ACC and prelimbic cortex, but decreasing their roles within the nodal vicinity. The importance of the somatosensory and ventral hippocampal regions was increased within the nodal vicinity. Here we discuss only those nodes for which several local metrics differed between the strains (both FDR-corrected and -uncorrected results).

The ACC had decreased clustering and local efficiency (closely related terms) but increased degree and participation. The decreased clustering and local efficiency indicate a decreased role within the nodal neighborhood, whereas the increased degree and participation imply an enhanced internodal role. Functionally, the rat ACC is a very diverse region: it participates in such cognitive functions as working memory for motor response, sequential learning and decision-making process.^29^ The abnormal functioning of this region could contribute to the cognitive deficits in depression.^30^ Also in rats the ACC is implicated in learning to discriminate visual stimuli on the basis of their association with reward.^31^ Lesion of this region in rats has pro-depressive effect, and high levels of stress and anxiety are associated with the reduction of the ACC activity.^32^ Taken together with our observation, it can be speculated that reduced local efficiency of the ACC in the NC strain in response to stress might similarly have a pro-depressive effect by affecting neighborhood connections. In contrast, its internodal role increased, possibly through enhancement of some long-range connections. The ACC has reciprocal amygdalar connections and participates in fear acquisition and expression: for example, ACC activation enhances these processes.^33^ In our data we have also observed the increased amygdalar strength in the NC rats (Figure 4). Taken together these data, we suggest that the bias to attend to negative information and deficit in cognitive and emotional integration characteristic for depression might be partially explained by the change in the ACC nodal role.

The prelimbic cortex, another DMN hub, had increased internodal role in the NC strain in response to stress, which was expressed as increased degree, strength and participation index. Similarly to the ACC, its local role reduced, which was reflected as a decrease in clustering coefficient. Our findings on the dorsal ACC and prelimbic cortex indicate a possible DMN hyperactivity, which is reported in depression patients^34, 35, 36, 37^ and is hypothesized to reflect an increased self-focus and tuning inward rather than to the external world as is manifested in depression symptoms. In our previous rs-fMRI study, we also demonstrated enhanced connectivity in the DMN, expressed as increased coupling between ACC and retrosplenial cortex and increased prefrontal–hippocampal connectivity in the NC compared to the PC rats.^38^ Another study on rats under chronic stress exposure had a similar finding of the DMN hyperconnectivity.^21^

In addition, the prelimbic activity is necessary for fear expression (measured as freezing duration).^33^ Earlier it has been demonstrated that the NC rats have increased fear expression (freezing behavior) in response to a tone formerly predictive of electric shock, and fail to show fear decrement during extinction—quite the contrary, they increase the level of freezing over the sessions.^39^ Similarly to the ACC, the prelimbic cortex has reciprocal connections with the amygdala, and the prelimbic–amygdalar pathway is crucial for fear memory encoding and fear expression.^33, 40^ We suggest that the enhanced prelimbic activity in the NC rats might contribute to increased fear expression.

Two other regions displaying increased local efficiency in the NC strain were the ventral hippocampus and somatosensory cortex. The ventral hippocampus encodes emotional and reward-relevant contents compared with spatially relevant information coded in the dorsal part.^41, 42^ Further studies are required to understand the possible implications of the differential functioning of these two regions and the meaning of our observations.

### Limitations

An anesthesia/sedation is required for the rodent scanning due to methodological and ethical concerns. As a general anesthesia can affect the brain connectivity, thus posing a challenge for interpretation, we used sedation and administered medetomidine, which acts as an agonist of α2-adrenoreceptors. This type of sedation allows recapitulation of the major features of brain networks in conscious states, that is, the functional connectivity maps analogous to humans.^24, 43^ We controlled the depth of sedation by measuring and recording the physiological (respiratory and cardiac) signal. As these signals can confound the BOLD signal, they were filtered out at the pre-processing step.

The second fMRI measurement included a lower number of rats (11 NC rats and 8 PC rats) than the first measurement because of the technical defects in the coil (the coil elements had to be replaced). Therefore, we had to exclude the distorted data from the final analysis. However, the final group sizes are typical of those used in rodent fMRI experiments^44, 45, 46^ and are judged to be sufficient for this hypothesis-generating study. The results provide specific hypotheses that can be tested in other studies.

## Conclusion

On the basis of our results, we suggest that transition from a high-risk endophenotype to a treatment-resistant depressive-like state is accompanied by increased internodal role of such DMN hubs, as the dorsal ACC and prelimbic cortex, and their reduced local efficiency. The latter might have a pro-depressive effect by affecting the neighborhood connections of these regions, whereas increased internodal role after stress could be because of the enhancement of some of their long-range connections, given their connectivity with the amygdala and other DMN hubs, which could create a bias to attend to negative information characteristic for depression. We suggest that these imaging endophenotypes could serve as a translational target for testing new antidepressants against TRD in a preclinical setting.
