# Supplementary material for: Brain network reorganization differs in response to stress in rats genetically predisposed to depression and stress-resilient rats
Source: Transl Psychiatry. 2016 Dec 6;6(12):e970–. doi: 10.1038/tp.2016.233 (PMC5315561; doi:10.1038/tp.2016.233)
Supplement: Supplementary Material S1 [file tp2016233x1.doc]

**Supplemental Material S1.**

Comparison of area-under-the-curve values for global and local parameters between the response to stress (behavioral test) in the negative cognitive state (NC) and positive cognitive state (PC) rat strains. The response was calculated as difference (delta) between the 2^nd^ and 1^st^ fMRI measurement for each strain, and two-sample T-test was used to compare these responses between the strains. The vertical blue line defines statistically significant values (p<0.05), the red line signifies values surviving false discovery rate correction (q<0.1). The Y-axis in the plots displaying changes in global parameters defines each global parameter; the Y-axis in the plots displaying changes in local parameters defines brain region (see explanation of abbreviations in the legend for Fig. 2). The X-axis in all plots defines T-statistic values.

**Global parameters**

Abbreviations: *g_Eglob_norm* - global efficiency; *g_Eloc_norm* - local efficiency; *g_cpl_norm* - characteristic path length; *g_swi_norm* - small-worldness index; *g_cc_norm* - global clustering coefficient.

**Local parameters**

**Path length**

**Degree**

**Strength**

**Betweenness centrality**

**Local clustering coefficient**

**Local efficiency**

**LEGE**

**Participation index**
