# Supplementary material for: Brain network reorganization differs in response to stress in rats genetically predisposed to depression and stress-resilient rats
Source: Transl Psychiatry. 2016 Dec 6;6(12):e970–. doi: 10.1038/tp.2016.233 (PMC5315561; doi:10.1038/tp.2016.233)
Supplement: Supplementary Material S2 [file tp2016233x2.doc]

**Supplemental Material S2.**

Before conducting parametric statistical tests we checked whether our data are normally distributed within the groups (required for F- and T-tests). Lilliefor’s test (a variant of Kolmogorov-Smiornov test) didn’t show significant deviation from normal distribution for the area under the curve of any global network metrics (p<0.05, uncorrected). Betweenness centrality was the only local metric which showed significant deviations (p<0.05, FDR-corrected) for very few regions, which were not included in the further discussion. Furthermore the data were checked for equal variances across groups (required for F-Tests) by a Brown-Forsyth test. There were no significant differences in group variances (p<0.05, uncorrected) for any global or local metrics.

The effect of the (1) time point (the 1st fMRI measurement versus the 2nd fMRI measurement), (2) the group (the PC group versus the NC group) and the interaction between (1) and (2) on the global and local parameters calculated by two-way repeated measurements ANOVA. The Y-axis in the plots display F-statistic values; one asterisk (*) indicates statistically significant changes (p<0.05), two vertical asterisks indicate the significant values surviving false discovery rate correction (q<0.1). The color coding is identical for the global and local parameters plots (see the legend at global parameters plot). The explanation of the abbreviations for the brain regions is the same as in the legend for Fig. 2.

**Global parameters**

Abbreviations: *g_Eglob_norm* - global efficiency; *g_Eloc_norm* - local efficiency; *g_cpl_norm* - characteristic path length; *g_swi_norm* - small-worldness index; *g_cc_norm* - global clustering coefficient.

**Local parameters**

**Path length**

**Degree**

**Strength**

**Betweenness centrality**

**Local clustering coefficient**

**Local efficiency**

**LEGE**

**Participation index**
